# Biomedical Frontiers with PVA–chitosan/lignin@CdZnO multifunctional hydrogel

**DOI:** 10.1039/d5ra02958a

**Published:** 2025-09-16

**Authors:** Hanzla Abdullah, Syed Ali Raza Naqvi, Fei Xiao, Muhammad Shahid Nazir, Yimin Sun, Aisha Rafique, Nadeem Ahmed Lodhi, Sadaf Ul Hassan

**Affiliations:** a Department of Chemistry, Government College University Faisalabad Faisalabad-38000 Pakistan; b Key Laboratory of Material Chemistry for Energy Conversion and Storage, Ministry of Education, School of Chemistry and Chemical Engineering, Huazhong University of Science & Technology Wuhan 430074 PR China; c Department of Chemistry, COMSATS University Islamabad Lahore Campus 54300 Lahore Pakistan; d Hubei Key Laboratory of Plasma Chemistry and Advanced Materials, School of Materials Science and Engineering, Wuhan Institute of Technology Wuhan 430205 China; e Isotope Production Division, Pakistan Institute of Nuclear Science and Technology (PINSTECH) P. O. Nilore Islamabad Pakistan

## Abstract

A nanoparticle-based hydrogel was synthesized and evaluated for advanced biomedical applications including antibacterial, anticancer and wound healing functionalities. The hydrogel was fabricated using polyvinyl alcohol (PVA), chitosan and lignin-mediated nanocomposites as the primary components. The resultant nanocomposites and hydrogel were examined using UV-visible spectroscopy, thermal gravimetric analysis, Fourier transform infrared spectroscopy and XRD techniques to confirm their composition and structural integrity. In the context of antibacterial studies, the PVA–chitosan/lignin@CdZnO (PVA-CS/L@CdZnO) hydrogel exhibited superior bactericidal activity compared with CdZnO and L@CdZnO nanocomposites, effectively inhibiting *Staphylococcus aureus* (*S. aureus*), *Bacillus subtilis* (*B. subtilis*), and *Escherichia coli* (*E. coli*). Antioxidant assays showed remarkable efficacy, with 89% of DPPH (2,2-diphenyl-1-picrylhydrazyl) activity and a reducing power absorbance of 0.68. Furthermore, in wound healing studies conducted on a rat model, the hydrogel achieved 91% wound closure within 10 days, significantly surpassing the 61% closure observed in the control group. Furthermore, against MCF-7 breast cancer cell lines, the hydrogel demonstrated 50% anticancer efficacy, showcasing its potential in oncological applications. These results underscore the multifunctional potential of the PVA–CS/L@CdZnO hydrogel, establishing it as an attractive choice for a wide range of biological applications, including antibacterial, antioxidant, anticancer, and wound healing therapies.

## Abstract 2

A nanoparticle-loaded hydrogel was prepared and evaluated for advanced biomedical applications including antibacterial, anticancer and wound healing functionalities.

## Graphical abstract

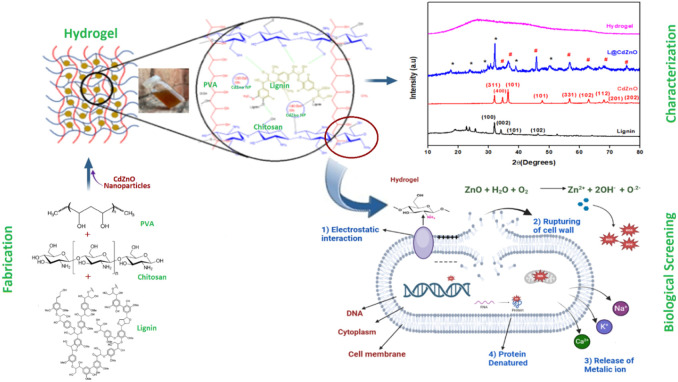

## 1 Introduction

A wound is a disturbance of the anatomical structure and functional continuity of the surrounding tissues. Wound healing is a complicated biological procedure that involves the restoration of tissue integrity. Physiologically, it may be divided into four separate phases: hemostasis, inflammation, proliferation, and tissue remodeling. It is influenced by a variety of elements, including interactions between the extracellular matrix, different kinds of cells, and growth hormones.^1^ The human skin has an intrinsic capacity to encourage self-regeneration after injury; however, this capability can be hampered in certain circumstances, like substantial skin loss, severe burns, chronic wounds, non-healing ulcers, and diabetes. Traditional medicines are becoming more prevalent in a variety of locations, including Africa, Asia, and Latin America, which help to improve healthcare accessibility. Traditional medicines provide primary care for around 80% of the population in Asia and Africa, while they account for 40% of the total healthcare practices in China.^2^ Conventional methods such as maggot debridement therapy, larval wound therapy, and herbal-derived substances have been widely employed for wound healing. Aside from their varied applications, all of these traditional treatments have limitations such as the onset of allergic responses, an unclear mechanism of action, the danger of infection, the rapid drying of the wound area, and batch-to-batch fluctuation, among others.^3^ Aside from that, many medications have been examined for wound healing, such as glucocorticoids,^4^ nonsteroidal anti-inflammatory drugs,^5^ and chemotherapeutic drugs,^6^ but due to the reduced oxygen flow at the wound when using these drugs, a slow healing process has been recorded. This leads to the exploration of cost-effective and more efficient strategies to heal wounds, especially in the case of diabetic foot wounds.

Nanotechnology has enabled the development of innovative wound-healing strategies, which have been proven to be cost-effective and efficient.^7^ In this regard, due to their biodegradability, biocompatibility, and tissue regeneration, more attention has been focused on hydrogels in the last few years.^8^ In recent research studies, a variety of hydrogels for different kinds of applications have been prepared.^9–12^ Hydrogels have been prepared using nanoparticles and macromolecules (hybrid hydrogels) according to the environmental conditions. The structure of hybrid hydrogels is temperature sensitive. Moreover, because of the presence of cations and anions in the hydrogel, environmental pH also influences the hydrogel structure of hybrid hydrogels. The term hybrid hydrogel refers to a structurally integrated network formed by combining three distinct biopolymeric components: cationic starch, carrageenan, and sodium alginate. Unlike conventional hydrogels composed of single or binary polysaccharide systems, the hybrid hydrogel described herein utilizes the complementary charge characteristics of its constituents to achieve enhanced physicochemical performance.^13^ Hydrogels are three-dimensional polymeric networks that have been effectively employed in a variety of biological applications. They function as a replacement extracellular matrix, creating a wet environment that promotes wound healing.^14^ Hydrogels are widely used in wound healing applications because they are similar to the original extracellular matrix (ECM) and may provide a moist environment. PVA is one of the most common and oldest synthetic polymers used for wound dressings, wound care, and drug delivery systems.^15^ The hydrogel's strength can be enhanced by the water-soluble, biocompatible, and biodegradable polymer PVA.^16^ The only downside of hydrogels is their weak mechanical stability when swollen. This disadvantage has been overcome by including a composite or hybrid hydrogel system containing more than one polymer in the dressing composition.^17^ Synthetic polymers may have limited strength and hydrophobic properties, which limit their applicability. Thus, natural polymers such as chitosan, which is formed by the hydrolysis of chitin's aminoacyl groups,^18^ have been utilized to repair wounds. Chitosan is a natural polysaccharide derived from chitin, a biopolymer found in the exoskeleton of crustaceans. This compound not only facilitates the formation of hydrogen bonds with PVA but also enhances the mechanical properties of the hydrogel through electrostatic interactions resulting from dynamic covalent bonds between the amino and carboxyl groups in chitosan.^19^ Despite having strong antibacterial and biocompatible qualities, the PVA–chitosan composite hydrogel's limited mechanical strength makes it unsuitable for use as a wound dressing. Moreover, the PVA–chitosan composite hydrogel does not meet the criteria necessary to function as an environmental conditioner that accelerates the wound healing process.^20^ The introduction of lignin in the PVA–chitosan hydrogel increases the mechanical strength, and because of its antioxidant and free radical scavenging qualities, the lignin–chitosan–PVA composite hydrogel speeds up healing in mouse skin wound therapy.^21^ Lignin, a polyphenolic polymer abundant in nature, contains numerous functional groups that enable hydrogen bonding and electrostatic interactions, thus enhancing the mechanical properties of composite films and hydrogels. Additionally, lignin is effective for modifying inorganic nanomaterials.^22^

Nano-reinforced multifunctional hydrogels represent a possible paradigm change in the area of wound healing. Biologically active nanoparticles, especially antibacterial and anti-inflammatory nanoparticles (NPs), in combination with biopolymers used in wound dressings, can promote the faster healing of both acute and chronic wounds.^23,24^ Metal oxide nanoparticles like CuO, ZnO, MgO, FeO, and CaO are considered to have potential antibacterial activity. It has also been reported that the formation of ZnO NPs and minocycline hydrogel composites significantly enhances the therapeutic and repairing abilities for gingival tissues.^25^ Similarly, cadmium oxide (CdO) nanoparticles, because of their antimicrobial qualities, can also be used to develop more valuable composite materials for better biological applications.^26^ Another report describes that cadmium (Cd) has the potential to kill cancer cells.^27^ Typical reported data reveal that each of PVA, chitosan, lignin, and CdZnO nanomaterials offer valuable biomedical potential. However, alongside their advantageous applications, it is revealed in literature that when these materials are used individually or in binary-combination fashions, they show poor mechanical strength, limited bioactivity, hydrophobicity, agglomeration and cytotoxicity risks.^28^ To overcome these challenges, in this research work, we developed a multifunctional hydrogel by integrating all four components, leveraging their complementary properties: PVA provides flexibility, chitosan adds antibacterial activity, lignin enhances the antioxidant capacity and mechanical strength, and CdZnO nanoparticles contribute antimicrobial and anticancer effects. This hybrid matrix, it is hypothesized, will ensure the improvement in biocompatibility and biomedical activities. The fabricated materials were characterized by using UV-vis, FT-IR, TGA, DSC, and XRD techniques. Furthermore, the DPPH free radical scavenging assay and reducing power assay were used to measure the hydrogel's antioxidant activity, while the well diffusion method was used for evaluating its antibacterial activity.

## 2 Materials and methods

### 2.1 Chemicals

Zinc chloride (ZnCl_2_), cadmium chloride (CdCl_2_), and sodium hydroxide (NaOH) were obtained from Sigma-Aldrich and used without further purification. PVA (*M*_w_ = 124–146 kg mol^−1^, 95% degree of hydrolysis) and 2,2-diphenyl-1-picrylhydrazyl (DPPH) free radical (*M*_w_ = 394.32 g mol^−1^) were also supplied by Sigma-Aldrich and used as received. Chitosan, a low molecular weight product, was purchased from Aldrich. Kraft lignin, a purified commercial polymer, was obtained from Aldrich. Throughout the experiment, ultra-filtered deionized (DI) water was used.

### 2.2 Preparation of CdZnO NPs

A 1% PVP solution was prepared by dissolving 1 g of PVP in 99 mL of distilled water. Then, 10 mL of this solution was mixed with 2.87 g of ZnCl_2_ in 100 mL of distilled water and stirred for 30 minutes. Subsequently, 0.056 g of CdCl_2_ was added to the mixture, and stirring was continued for 1 hour. A NaOH solution was then added dropwise, leading to the formation of white precipitates. The resulting precipitates were washed thoroughly with methanol and filtered. To eliminate any residual impurities, the filtrate was dried in an oven at 80 °C for 12 hours. After drying, the precipitates were ground into a uniform powder and heated for 4 hours at 550 °C in a muffle furnace.^29^

### 2.3 Synthesis of L@CdZnO

**Scheme 1:**
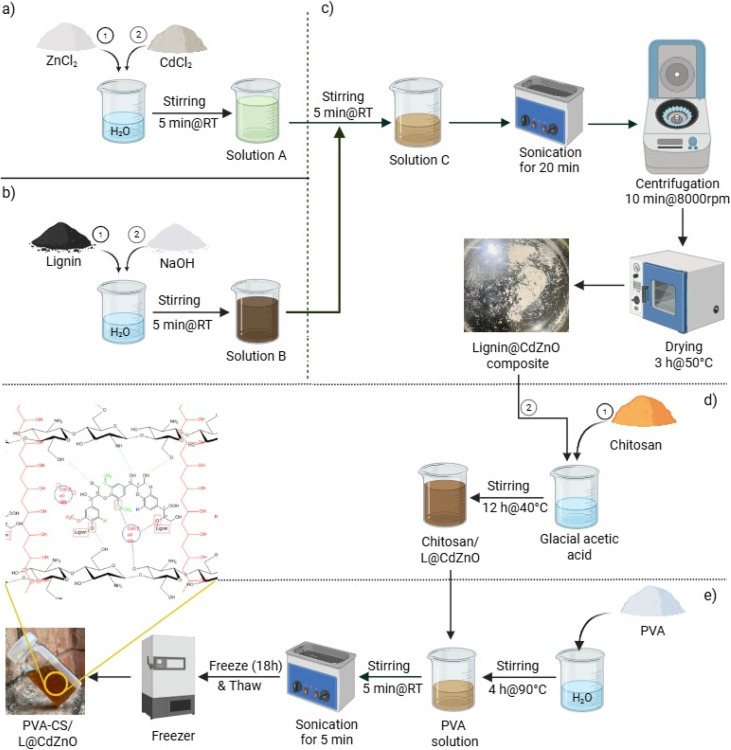
Fabrication of the PVA–CS/L@CdZnO hydrogel: (a) synthesis of solution A by mixing CdCl_2_ and ZnCl_2_; (b) synthesis of solution B by mixing lignin and NaOH; (c) formation of the L@CdZnO composite by mixing solutions A and B; (d) synthesis of CS/L@CdZnO by mixing chitosan in acetic acid and L@CdZnO and (e) finally, the fabrication of the hydrogel by mixing the PVA solution with CS/L@CdZnO to form PVA–CS/L@CdZnO.

The L@CdZnO nanocomposite was synthesized using the probe sonication method. In the formulation of solution A, 0.0025 M (0.458 g) CdCl_2_ and 0.0025 M (0.340 g) ZnCl_2_ were dissolved in 70 mL of deionized water and stirred until homogeneity was achieved. Solution B was prepared by dissolving 0.013 M (0.6 g) lignin and 0.25 M (0.3 g) NaOH in 30 mL of DI water and stirring constantly until lignin was completely dissolved. Solution B was then gradually introduced into solution A, followed by stirring for an additional 15 minutes. The resultant solution underwent probe sonication for 20 minutes at 60% amplitudes with a 5-second pulse interval. Centrifugation at 8000 rpm for 10 minutes was used to separate the final product, which was then dried in an oven set at 100 °C for 3 hours.^29^ The scheme for the fabrication of L@CdZnO is shown in Scheme 1(c).

### 2.4 Fabrication of the PVA–CS/L@CdZnO hydrogel

To prepare the PVA–CS/L@CdZnO nanocomposite-based hydrogel, PVA (5 g) was dissolved in 20 mL of DI water under continuous magnetic stirring at 90 °C for 4 hours. Separately, 0.5 g of chitosan was dissolved in 10 mL of glacial acetic acid with magnetic stirring at 40 °C for 12 hours until a homogeneous solution formed. Afterward, 0.05 g of the L@CdZnO nanocomposite was added to the chitosan solution and thoroughly mixed. Both solutions were combined and subjected to sonication for 5 minutes to ensure uniform dispersion. The final mixture was poured into a Petri dish, covered with a Teflon lid, and left to solidify under controlled conditions.^30^ The detailed scheme for the fabrication of the PVA–CS/L@CdZnO hydrogel is shown in Scheme 1(a–e).

### 2.5 Characterizations

The synthesized materials were thoroughly characterized using Fourier transform infrared (FT-IR) spectroscopy, UV-visible spectroscopy, X-ray diffraction (XRD), and thermogravimetric analysis (TGA) following the reported protocols.^31^ FT-IR analysis was performed using a Thermo-Nicolet 6700 spectrometer (USA) to identify the functional groups present in the CdZnO nanoparticles (NPs), L@CdZnO NPs, and PVA–CS/L@CdZnO hydrogel, covering a spectral range from 400 to 4000 cm^−1^. The optical properties of the CdZnO NPs, L@CdZnO, and PVA–CS/L@CdZnO hydrogel were investigated using UV-visible spectroscopy with a LAMBDA 25 spectrophotometer, scanning in the wavelength range of 200–800 nm. The phase composition and crystalline structure were determined *via* XRD utilizing CuKα radiation in a 2*θ* range from 0° to 90°. To examine the thermal stability and decomposition behavior, TGA was conducted using an SDT Q600 instrument.

### 2.6 Antibacterial activity

The antibacterial activity of CdZnO, L@CdZnO, and PVA–CS/L@CdZnO hydrogel was assessed using a slightly modified version of the well diffusion method described previously.^32^ The Department of Microbiology at the GCUF provided one Gram-negative strain, *i.e.*, *E. coli*, and two Gram-positive strains, *i.e.*, *S. aureus* and *B. subtilis*, for the antibacterial evaluation. For the test medium, 3.8 g of Mueller–Hinton agar was dissolved in 100 mL of distilled water and sterilized in an autoclave at 121.7 °C for 20 minutes. Bacterial cultures were equally distributed on the surface of Petri dishes after the sterilised media had been added and allowed to solidify. Stock solutions of the test samples were prepared in dilute acetic acid, followed by serial dilutions of 3 mg mL^−1^, 1.5 mg mL^−1^, 0.75 mg mL^−1^, and 0.375 mg mL^−1^. Acetic acid (30 μg mL^−1^) was used as a negative control, while CFR 30 (30 μg mL^−1^) served as a positive control. Five wells were made in each plate, and 40 ppm from each dilution was added to the corresponding wells, with CFR 30 positioned at the center. The plates were incubated at 37 °C for 24 hours, after which the bacterial growth was measured as zone of inhibition (ZOI) in mm. Triplicate measurements were performed for each sample to ensure the accuracy and precision of the results.

### 2.7 Antioxidant study

#### 2.7.1 DPPH free radical scavenging activity

DPPH is a stable free radical frequently used to evaluate the radical scavenging activity of the antioxidant components. The DPPH free radical scavenging activity of CdZnO, L@CdZnO, and the hydrogel was assessed by using a previously reported protocol with slight modifications.^33^ Briefly, methanol solutions of CdZnO, L@CdZnO, and PVA–CS/L@CdZnO hydrogel samples at varying concentrations (1000 μg, 500 μg, 250 μg, and 125 μg) were individually mixed with 4 mL of a 0.2 mM methanol DPPH free radical solution. After keeping the mixture in the dark for 30 minutes at room temperature, the absorbance was measured at 517 nm using methanol as a blank. To ensure the accuracy, assay was carried out three times. The DPPH free radical scavenging potential was measured by the following eqn (1):1
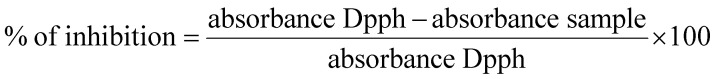
The DPPH radical scavenging activity increases with decreasing absorbance.

#### 2.7.2 Reducing power assay

The antioxidant activity of CdZnO, L@CdZnO, and the hydrogel was determined according to the previously reported methods.^34^ Specifically, 2.5 mL of the samples (CdZnO, L@CdZnO, and PVA–CS/L@CdZnO hydrogel) at varying concentrations (62.5 μg mL^−1^, 125 μg mL^−1^, 250 μg mL^−1^, 500 μg mL^−1^, and 1000 μg mL^−1^) was mixed with 2.5 mL of 200 mmol L^−1^ sodium phosphate (pH 6.6) and 2.5 mL of a potassium ferricyanide solution. The mixture was incubated at 50 °C for 20 minutes in an incubator. Next, 2.5 mL of 10% w/v trichloroacetic acid was added after incubation, and centrifugation was performed for 10 minutes at 650 rpm. Five millilitres of the obtained supernatant were taken and combined with 1.0 mL of 0.1% ferric chloride and 5 mL of distilled water. Ascorbic acid was used as the reference standard for measuring the final solution's absorbance at 700 nm. Greater absorption resulted in an increased reducing power.

### 2.8 Wound healing potential

The wound healing potential of the prepared hydrogels was evaluated in white Albino rats weighing 250–300 g. A total of four rats were used in the study, and they were divided into two groups: Group 1 served as the control group, while Group 2 received the treatment. The animal study for wound healing was approved by the Department of Pharmaceutics, Government College University, Faisalabad, Pakistan. Briefly, to induce wounds, the rats were anesthetized with chloroform, and their backs were shaved using a hair trimmer. Using sterile surgical tools, a full-thickness excisional incision with a 5-mm diameter was created on the dorsal surface. The treatment was then directly applied to the wounds. Images of the wounds were captured on days 0, 2, 4, 6, 8, and 10 to assess the percentage reduction in the wound size for each group.^35^

### 2.9 Anticancer activity

The 3-(4,5-dimethylthiazol-2-yl)-2,5-diphenyltetrazolium bromide (MTT) assay protocol was carried out to examine the cytotoxicity of the prepared CdZnO, L@CdZnO, and PVA–CS/L@CdZnO hydrogel.^36^ The cancerous cell line MCF7 (breast cancer) was collected from the Microbiology Department, GCUF. The following eqn (2) and (3) were used to estimate the viability and inhibition percentage, respectively.2
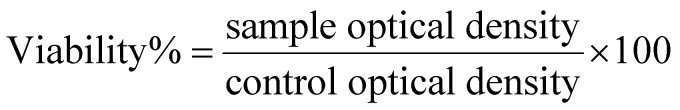

3 Inhibition% = 100 − viability%

## 3 Results and discussion

### 3.1 UV-visible analysis

**Fig. 1:**
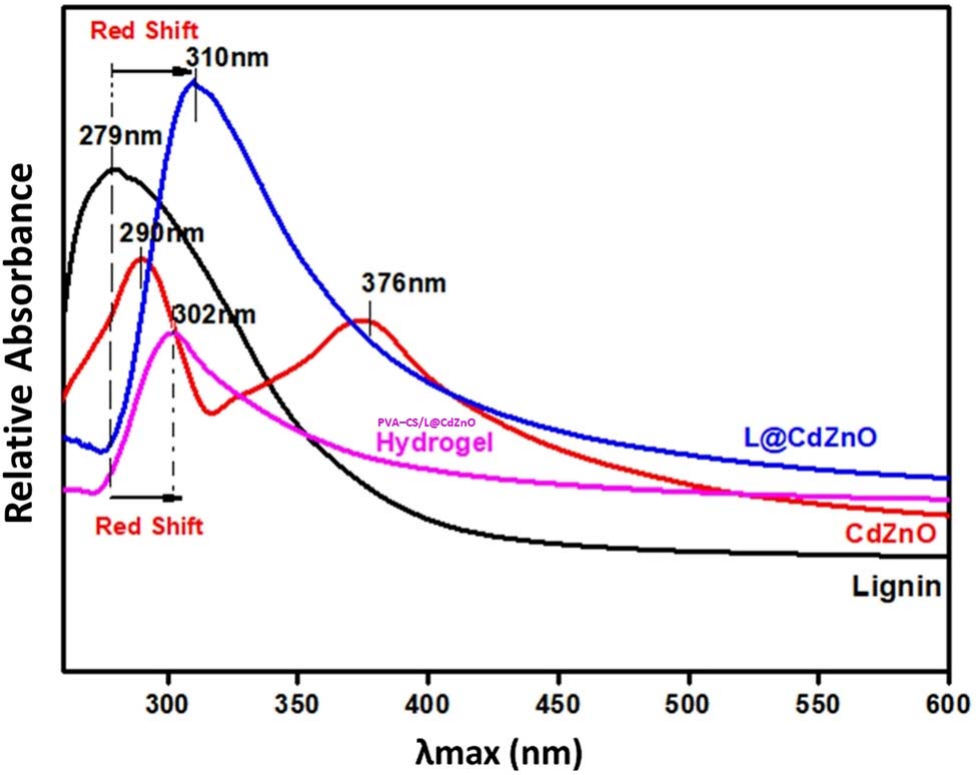
UV-visible spectra of lignin, CdZnO NPs, L@CdZnO, and the PVA–CS/L@CdZnO hydrogel.

The UV-visible spectra of lignin, CdZnO, L@CdZnO, and the hydrogel are illustrated in Fig. 1. Lignin shows the maximum absorption at 279 nm, which is its characteristic UV-peak due to the electronic conjugated system established by benzene moieties.^29^ CdZnO NPs display broad peaks at 290 nm and 376 nm, which are attributed to the presence of CdO and ZnO nanoparticles, respectively.

Interestingly, the L@CdZnO nanocomposite shows a peak at 310 nm (*λ*_max_), which indicates a red shift with respect to lignin, which typically absorbs at 279 nm (*λ*_max_). This could be explained as follows: when lignin forms a composite with CdZnO, the localized electronic cloud of CdZnO appears to be delocalized. This results in the reduction in the HOMO and LUMO gap, which allows more frequent π–π* electronic transitions at low energy (310 nm wavelength). Similarly, the PVA–CS/L@CdZnO hydrogel shows the maximum absorption (*λ*_max_) at 302 nm. Firstly, this is attributed to the formation of the PVA–CS/L@CdZnO hydrogel, as it absorbs at a different wavelength compared to the L@CdZnO composite (310 nm). Secondly, the slight decrease in the absorbance is, typically, due to the polyvinyl alcohol and polysaccharide structural hindrance to the frequent π–π* electronic transitions, which we observed in the case of the L@CdZnO composite.

### 3.2 FT-IR analysis

**Fig. 2:**
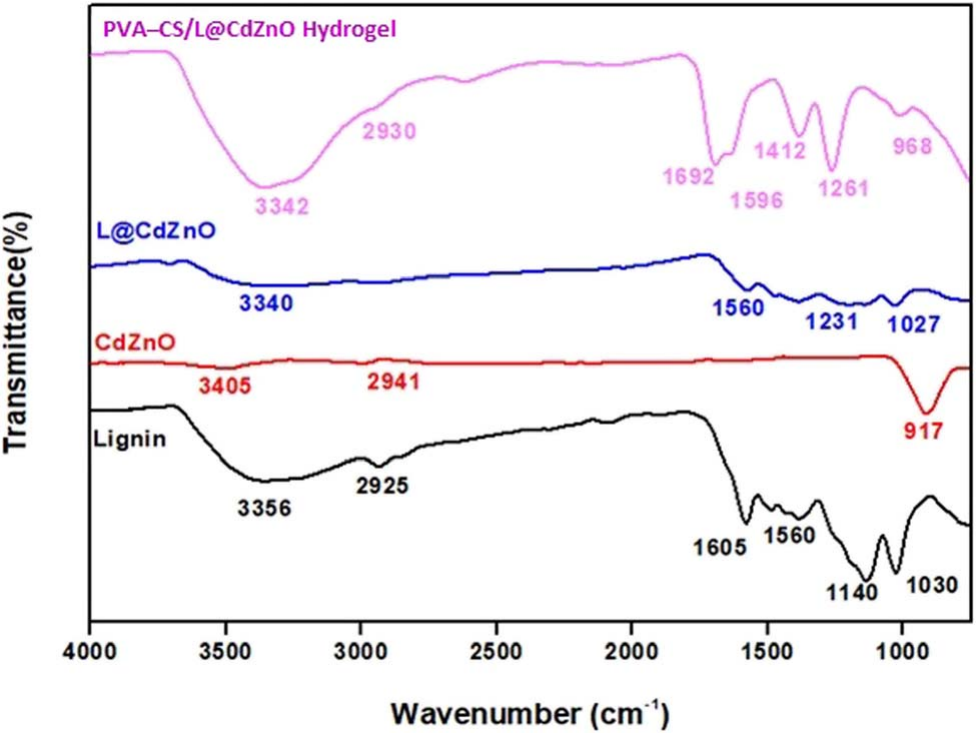
FT-IR spectra of lignin, CdZnO NPs, L@CdZnO, and the PVA–CS/L@CdZnO hydrogel.

FT-IR analysis was used to investigate the molecular structure, chemical bonding, and functional groups present in lignin, CdZnO, L@CdZnO, and PVA–CS/L@CdZnO hydrogel, as shown in Fig. 2, in the spectral range from 4000 cm^−1^ to 500 cm^−1^. The FT-IR spectrum of lignin reveals characteristic peaks at 3356 cm^−1^ (–OH stretching), 2925 cm^−1^ (C–H stretching of methyl and methylene groups), 1605 cm^−1^ (C=O stretching from conjugation to the aromatic ring), 1560 cm^−1^ (C=C stretching from aromatic skeleton vibrations), and 1140 and 1030 cm^−1^ for C–O stretching and deformation, respectively.

The FT-IR spectrum of CdZnO shows peaks at 3405 cm^−1^, 2941 cm^−1^, and 917 cm^−1^, attributed to the C–H stretching, asymmetric and symmetric stretching of the C–H, and C=O of the carboxylic group, respectively.^37^ The FT-IR spectrum of the L@CdZnO composite retains all the characteristic peaks of lignin, suggesting that the chemical structure of lignin remains largely unchanged. This indicates the successful integration of CdZnO nanocomposite into the lignin matrix without significant alteration in its molecular structure.^38^ The FT-IR spectrum of the hydrogel reveals a broad band at 3342 cm^−1^, corresponding to O–H stretching from both aromatic and aliphatic groups. The sharp peak around 1692 cm^−1^ represents the characteristic band of lignin. The appearance of a new peak at 1596 cm^−1^ suggests the formation of imine bonds due to cross-linking. The broad absorption band observed in all spectra around 3340–3405 cm^−1^ corresponds to overlapping –OH and –NH_2_ stretching vibrations, typical of lignin and chitosan components. In the hydrogel spectrum, a new and distinct band appears at 1596 cm^−1^, which is not present in the spectra of individual components. This band is attributed to the C=N stretching vibration, indicating the formation of imine (Schiff base) linkages. Imine bonds typically arise from a condensation reaction between a primary amine (–NH_2_) and a carbonyl group (aldehyde or ketone, –C=O). In this system, the –NH_2_ groups are contributed by chitosan, a biopolymer known for its primary amine functionality. The carbonyl groups are likely derived from oxidized lignin, which contains aldehyde and ketone moieties as a result of depolymerization or chemical modification. This type of crosslinking enhances the structural integrity and stability of the hydrogel network. The imine bond is formed by the condensation reaction between the amine group from chitosan and aldehyde or the ketone group from the oxidized or cross-linked lignin PVA.^39^ In addition to the FT-IR peaks obtained in case of L@CdZnO IR spectra, the hydrogen bonding peak, likely between the hydroxyl and carbonyl groups in lignin and chitosan, confirms the formation of PVA-CS/L@CdZnO hydrogel. Additionally, the peak at 968 cm^−1^, attributed to CdZnO NPs, further verifies the successful synthesis of the hydrogel.

### 3.3 TGA analysis

**Fig. 3:**
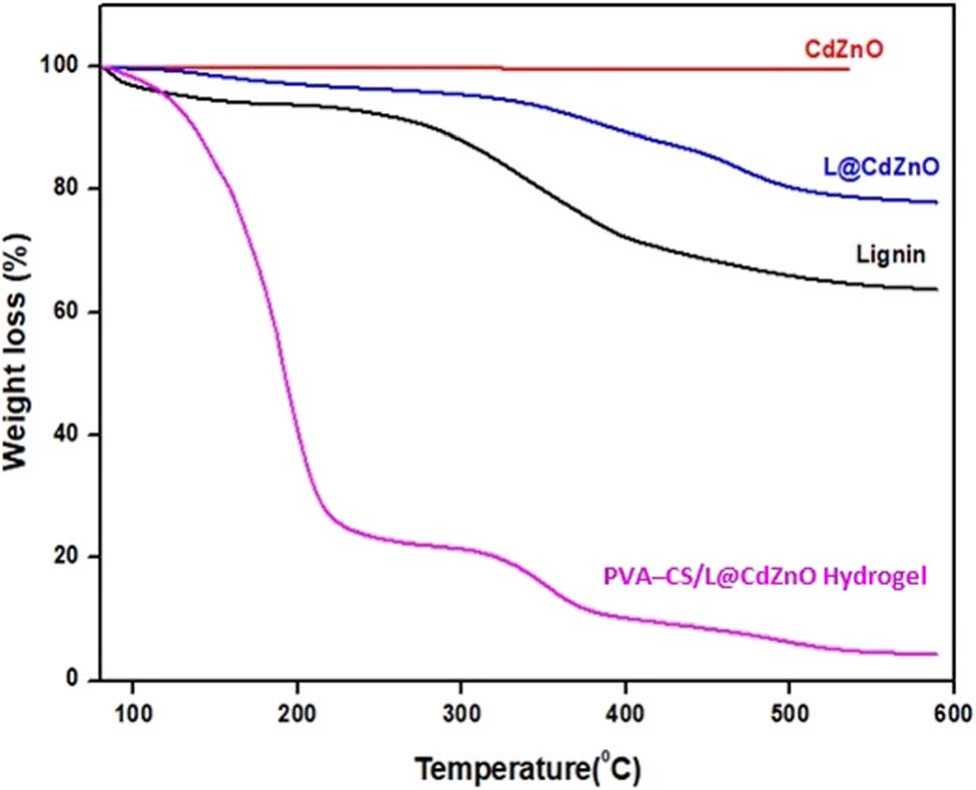
TGA spectra of lignin, CdZnO NPs, L@CdZnO, and the PVA–CS/L@CdZnO hydrogel.

The thermal stability of lignin, CdZnO NPs, L@CdZnO, and PVA–CS/L@CdZnO hydrogel was assessed through TGA, as shown in Fig. 3. The TGA curve of CdZnO showed a minimal weight loss of 2%, indicating a negligible amount of bound water or impurities, confirming the high purity of the nanocomposite. The thermal degradation of lignin occurred in three stages. The evaporation of absorbed and bonded water was the cause of the initial 6.2% weight loss between 80 and 160 °C. The primary cause of the 26% weight loss between 160 and 470 °C was the breakdown of lignin into volatile chemicals, while the slower degradation in the third stage, from 470 to 600 °C, produced char, leaving approximately 63% of the sample remaining at 600 °C.

The TGA curve of L@CdZnO also comprised three stages. In the first stage, due to the elimination of moisture, there was a 3.81% weight loss between 80 and 130 °C. In the second stage, between 250 and 400 °C, lignin decomposed, with a reduced weight loss of 9.02%, attributed to the incorporation of nanoparticles, which enhanced thermal stability. The final stage showed continued slow degradation, leaving about 76% of the sample remaining at 600 °C. The hydrogel's TGA curve also showed degradation in three stages. In the first stage from 30 to 120 °C, the major weight loss (74%) was due to the evaporation of water and acetic acid in the hydrogel. In the second stage from 200 to 350 °C, the decomposition of polyvinyl alcohol and chitosan resulted in a 13% weight loss. In the third stage, degradation ranged from 350 to 550 °C, and weight loss (5.96%) occurred due to the degradation of the saccharide rings and the DE polymerization of the acylated and deacylated units, with approximately 4% of the sample remaining at 600 °C, as shown in Table 1. TGA was performed to assess the thermal stability of the PVA–CS/L@CdZnO hydrogel, a parameter of significance for biomedical applications. Thermal stability is a critical consideration in the context of sterilization procedures (such as autoclaving or dry heat treatment), storage conditions, and the material's *in vivo* performance, particularly where exposure to physiological or elevated temperatures may occur. These characteristics enhance the hydrogel's suitability for biomedical applications such as drug delivery systems, wound dressings, or implantable scaffolds, where predictable thermal behavior is essential.

**Table 1:** TGA results of lignin, CdZnO NPs, L@CdZnO, and the PVA–CS/L@CdZnO hydrogel

Material	*T*_onset_ (°C)	Peak 1		Peak 2		Peak 3		Residues at 600 °C
		*T*_max_ (°C)	Wt%	*T*_max_ (°C)	Wt%	*T*_max_ (°C)	Wt%	
Lignin	81	160	6.2	470	26	588	5	63
CdZnO	52	550	2	—	—	—	—	98
L@CdZnO	50	130	3.81	400	9.02	550	10	76
PVA–CS/L@CdZnO	29	120	74	350	13	550	5.96	4

### 3.4 XRD analysis

**Fig. 4:**
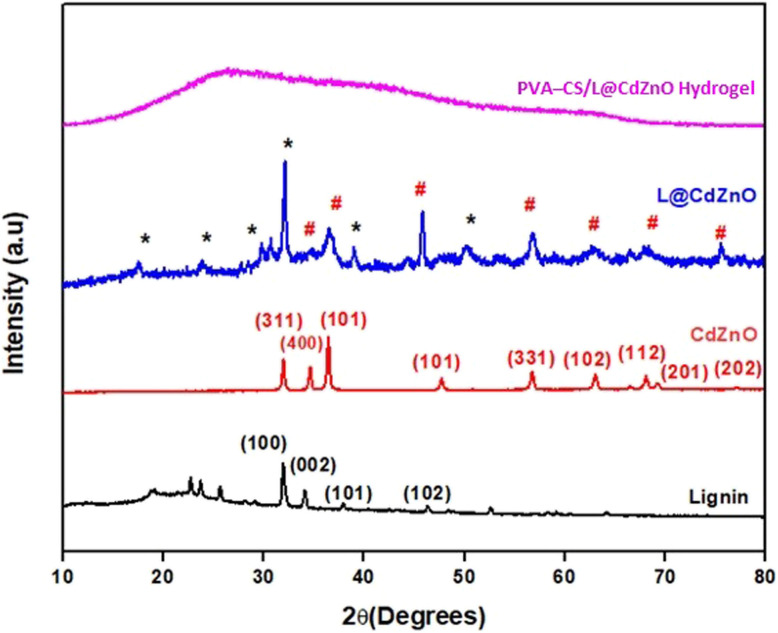
XRD spectra of lignin, CdZnO NPs, L@CdZnO, and the PVA–CS/L@CdZnO hydrogel.

The XRD spectra of lignin, CdZnO, L@CdZnO, and PVA–CS/L@CdZnO hydrogel show crystallographic structures, showing atomic arrangement in the octahedral configuration in Fig. 4. Lignin shows diffraction peaks at 31.87°, 34.16°, 37.99°, 46.27°, and 52.51°, corresponding to the diffraction planes 100, 002, 101, 102, and 110, respectively.^29^ Previous studies also confirm the diffraction peaks of lignin at these planes to confirm the purity of lignin 29. In the diffraction spectrum of the CdZnO, the sharp peaks at 2*θ* values of 32.16, 34.64, 47.65, and 56.72 correspond to (311), (400), (101), and (331), respectively, indicating the reflection planes of CdO NPs (JCPDS card no. 005-0628),^40^ and other peaks values such as 2*θ* values of 36.44, 63.02, 68.06, 69.34, and 77.02 are associated with (101), (102), (112), (201), and (202), respectively, reflection planes of ZnO NPs (JCPDS no. 75-1526).^41^ The average crystalline size of the CdZnO NPs was calculated by Debye–Scherer's formula, as shown in the following eqn (4):4
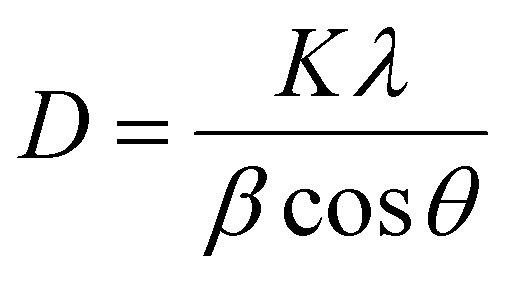
where *K* denotes the form factor (0.94), *λ* is the X-ray wavelength of radiation for CuKα (1.54056 Å), *β* is the full width at the half-maximum intensity (FWHM), *θ* is the Bragg diffraction angle, and *D* is the crystallite size. Based on this equation, the estimated average size of the CdZnO is 19.05 nm. The XRD spectrum of L@CdZnO was confirmed against the standard of lignin and CdZnO NPs. The reflection planes at 2*θ* values of 31.87 (100), 34.16 (002), 37.99 (101), and 46.27 (102) confirm the presence of lignin in the L@CdZnO.^29^

The remaining peaks at 2*θ* values of 32.16 (311), 34.64 (400), 47.65 (101), and 56.72 (331) can be related to the crystal planes of CdZnO NCs (JCPDS card no. 005-0628).^40^ The estimated mean particle size of the L@CdZnO nanocomposite is 24.61 nm. CdZnO and L@CdZnO particles exhibit hexagonal wurtzite shapes with the sizes of 19.05 nm and 24.61 nm, respectively, while the hydrogel displays an amorphous structure. Hydrogel formation was confirmed by the characteristic peaks of the PVA and chitosan when L@CdZnO was added to it. The two diffraction peaks at 20° and 22° are the characteristic peaks of PVA and chitosan, respectively.^42^ The hydrogel exhibits a broad peak at a 2*θ* value of 27°, indicating the presence of a significant amount of an amorphous material, likely lignin. The XRD pattern of the PVA–CS/L@CdZnO hydrogel confirms the absence of crystalline morphology of CdZnO NP, *i.e.*, a homogenous dispersion of CdZnO NP in PVA-CS/L hydrogel either through hydrogen bonding or electrostatic forces of attraction. This suggests that the presence of PVA and chitosan reduces the crystallinity of the L@CdZnO nanoparticles. This behavior is attributed to the significant hydrogen bonding among PVA, chitosan, and L@CdZnO, confirming the successful formulation of PVA–CS/L@CdZnO hydrogel.^43^

### 3.5 Antibacterial activity

**Fig. 5:**
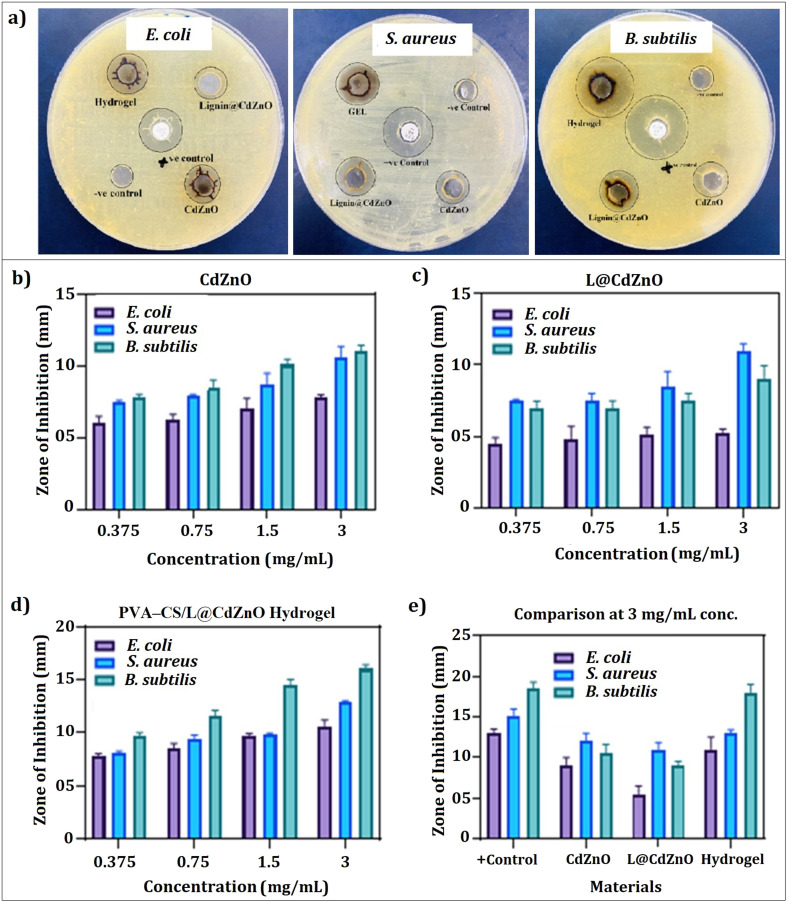
Antibacterial activity observed using the disc diffusion process (a), results of the antibacterial effect of CdZnO NPs (b), L@CdZnO (c), and the PVA–CS/L@CdZnO hydrogel composite (d) at different concentrations, and a comparative evaluation among all the materials of 3 mg mL^−1^ concentration using *E. coli*, *S. aureus* and *B. subtilis* bacterial strains (e).

The antibacterial activity of CdZnO, L@CdZnO and PVA–CS/L@CdZnO hydrogel was evaluated towards *S*. *aureus*, *B. subtilis*, and *E. coli* using the well diffusion method, as shown in Fig. 5. Results indicated that the nanocomposites at a concentration of 3 mg mL^−1^ expressed the largest zone of inhibition against all three kind of bacterium.

Specifically, CdZnO at 3 mg mL^−1^ achieved the highest ZOI against *S. aureus* (12 mm), *B. subtilis* (9.5 mm), and *E. coli* (9 mm), while the maximum ZOIs expressed by L@CdZnO were 11 mm, 9 mm, and 5 mm against *S. aureus*, *B. subtilis*, and *E. coli*, respectively. Promising antibacterial activity of PVA–CS hydrogel (without nanoparticles) has already been reported against *S. aureus* and *E. coli*.^30^ In our study, the PVA–CS/L@CdZnO hydrogel demonstrated the most significant antibacterial effect, with the ZOIs of 18 mm, 13 mm, and 11 mm against *B. subtilis*, *S. aureus*, and *E. coli*, respectively. Current reported studies also reveal the effectiveness of different hydrogels against bacterial infections, but we found quite encouraging results.^44^ The bactericidal effect of ZnO NPs is effective against Gram-positive as well as Gram-negative bacteria because of their increased surface area relative to microparticles, which enhances antibacterial efficacy.^45^ The CdZnO exerts antibacterial effects primarily by the release of metal ions and reactive oxygen species (ROS), which disrupt bacterial cell membranes and high protein transport and ultimately cause cell death.^37,46^ Furthermore, the antimicrobial potential of chitosan has also been reported in different formulations, particularly in acidic environments (pH < 6.5) where deacetylated C_2_ amino groups in chitosan become protonated and positively charged. These positive charges interact with the negatively charged microbial cell walls and membranes, leading to increased cell permeability, osmotic damage, and the leakage of cytoplasmic contents, including essential proteins and ions.^47,48^

**Fig. 6:**
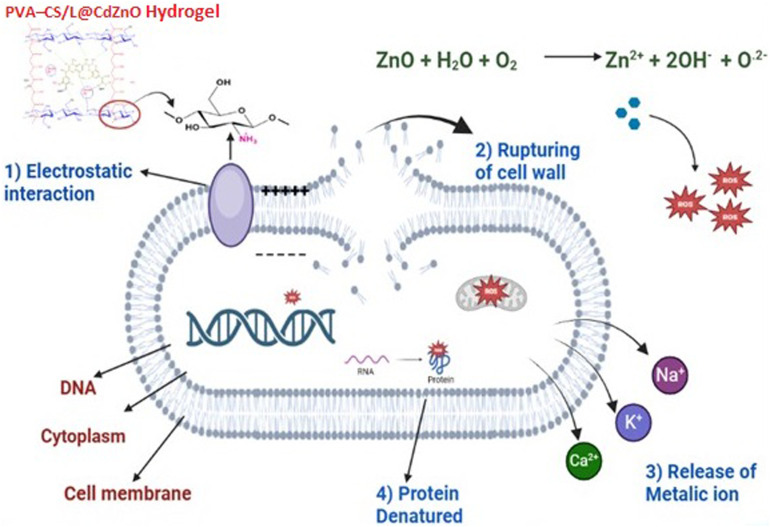
Antibacterial mechanistic action of the PVA–CS/L@CdZnO hydrogel.

In summary, our fabricated PVA–CS/L@CdZnO hydrogel demonstrated enhanced antibacterial efficacy due to the multiple antibacterial hydrogel components, which trigger the generation of ROS, and the release of metallic ions, dominantly Zn^2+^ and Cd^2+^ in biological system.^37^ Further, deacetylated C_2_ amino groups of chitosan molecules,^48^ and the presence of phenolic hydroxyl and methoxy groups on the surface of lignin also play effective role in antibacterial activities.^49^ These factors collectively contributed to the PVA–CS/L@CdZnO hydrogel's strong antibacterial potential. Hence, PVA–CS/L@CdZnO hydrogels are proposed to be used for the treatment of periodontal disease due to their inhibitory effects. The mechanistic interaction of the hydrogel composite with the bacterial cell wall and the resulting fate of the bacterial cell are shown in Fig. 6.

### 3.6 Antioxidant activities

#### 3.6.1 DPPH scavenging activity

**Fig. 7:**
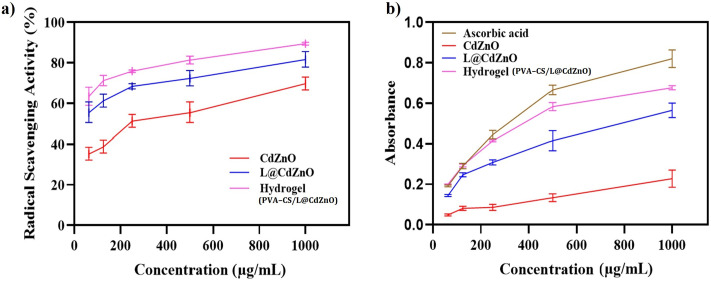
Antioxidant activity study: (a) DPPH scavenging activity of CdZnO NPs, L@CdZnO, and the PVA–CS/L@CdZnO hydrogel and (b) reducing power of ascorbic acid, CdZnO NPs, L@CdZnO, and the PVA–CS/L@CdZnO hydrogel composite.

The antioxidant activity of CdZnO, L@CdZnO, and PVA–CS/L@CdZnO hydrogel composite was assessed using the DPPH free radical scavenging activity, as shown in Fig. 7(a). This activity relies on the capacity of antioxidants to neutralize the stable free radical, DPPH, leading to a decrease in absorbance detectable by the UV spectrophotometer. Antioxidants change the purple color of DPPH into a yellow color. At a concentration of 1000 μg mL^−1^, the synthesized L@CdZnO had a better antioxidant activity of 81%, whereas CdZnO showed a higher reducing scavenging activity of 70% at 1000 μg mL^−1^. The PVA–CS/L@CdZnO hydrogel expressed the highest DPPH activity of 89% at 1000 μg mL^−1^. After modifying CdZnO with lignin, the DPPH scavenging activity increased from 70% to 80% at a 1000 μg mL^−1^ concentration. The highest DPPH scavenging activity was expressed by the hydrogel, which was formed *via* the fabrication of L@CdZnO onto PVA/CS. According to the formulation of the hydrogel, the increasing scavenging potential of PVA–CS/L@CdZnO hydrogel was likely due to the addition of chitosan with the antioxidant potential. The scavenging effect of chitosan is based on the free amino (NH_2_) groups' ability to bind with free radicals and form stable macromolecule radicals. After that, NH_2_ groups take up hydrogen ions from the mixture, producing ammonium (NH^3+^) groups.

#### 3.6.2 Reducing power assay

The reducing power assay, used to investigate the antioxidant activity, measures the reduction of ferric ions into ferrous ions in the presence of an antioxidant, resulting in a color shift from yellow to green or blue. This change is detected as an intense blue Prussian complex at 700 nm, with ascorbic acid serving as the standard. The CdZnO showed absorbance values of 0.053, 0.089, 0.097, 0.147, and 0.258, whereas L@CdZnO showed absorbance values of 0.141, 0.254, 0.317, 0.451 and 0.591. The hydrogel showed absorbance values of 0.201, 0.298, 0.419, 0.598, and 0.685, and ascorbic acid, which was used as the standard, had absorbance values of 0.195, 0.300, 0.460, 0.683, and 0.851, as shown in Fig. 7(b). The CdZnO showed slight antioxidant activity due to metal oxide nanoparticles. A study revealed that with increasing nanoparticle concentration, the reducing power also increases.^50^ However, when lignin was attached to the nanoparticles, the reducing power increased from 0.258 to 0.591 with increasing absorbance. Interestingly, the reducing potential of L@CdZnO was significantly higher than that of native lignin by several orders of magnitude.^51^ As was stated earlier, the phenolic hydroxyl groups are the primary component that governs the redox activity of lignin.^52^ The PVA–CS/L@CdZnO hydrogel showed a strong reducing power because it contains lignin and chitosan as additional factors. A prior investigation demonstrated the antioxidant effects of chitosan.^53^ Because chitosan chains may chelate ferrous ions and are therefore a source of antioxidants, they also contain active hydroxyl and amino groups, which give chitosan its antioxidant potential. Therefore, the PVA–CS/L@CdZnO hydrogel could be a novel candidate for antioxidant activities.

### 3.7 Wound healing potential

**Fig. 8:**
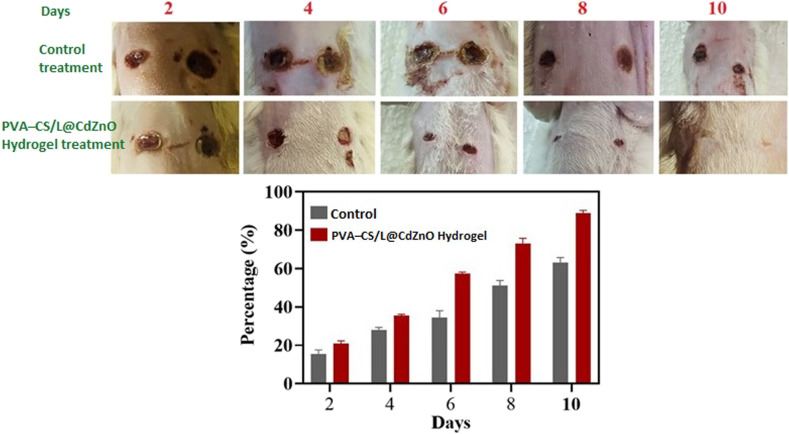
Quantitative wound healing analysis of PVA–CS/L@CdZnO hydrogel in comparison with positive cotrol drug in rats.

*In vivo* wound healing studies were conducted to assess the effectiveness of the PVA–CS/L@CdZnO hydrogel in accelerating wound closure. Fig. 8 shows the control group and the treatment group using the hydrogel, which adhered directly to the wounds without additional preparation. The gel-like consistency of the hydrogel created a protective layer over the wound, supporting antimicrobial defense. Results indicated that wounds treated with the hydrogel healed significantly faster than those in the control group. Observations noted early re-epithelialization and the formation of new blood vessels under the hydrogel, with complete wound closure by day 10. The statistical analysis highlighted a notable reduction in the healing time for hydrogel-treated wounds, with marked improvements observed on days 6, 8, and 10. Initially, both groups showed similar wound statuses up to day 3, but by day 6, the hydrogel-treated wounds demonstrated considerably faster healing, with complete closure by day 10. Wound healing typically progresses through the phases of hemostasis, PVA–CS/L@CdZnO inflammation, proliferation, and maturation. The enhanced healing observed in the hydrogel group was attributed to the active antioxidant components from the lignin and chitosan within the hydrogel matrix. Previous research supports the use of lignin-based hydrogels as effective antioxidant wound dressings, underscoring the hydrogel's potential as an advanced wound treatment option.

### 3.8 Anticancer activities

**Fig. 9:**
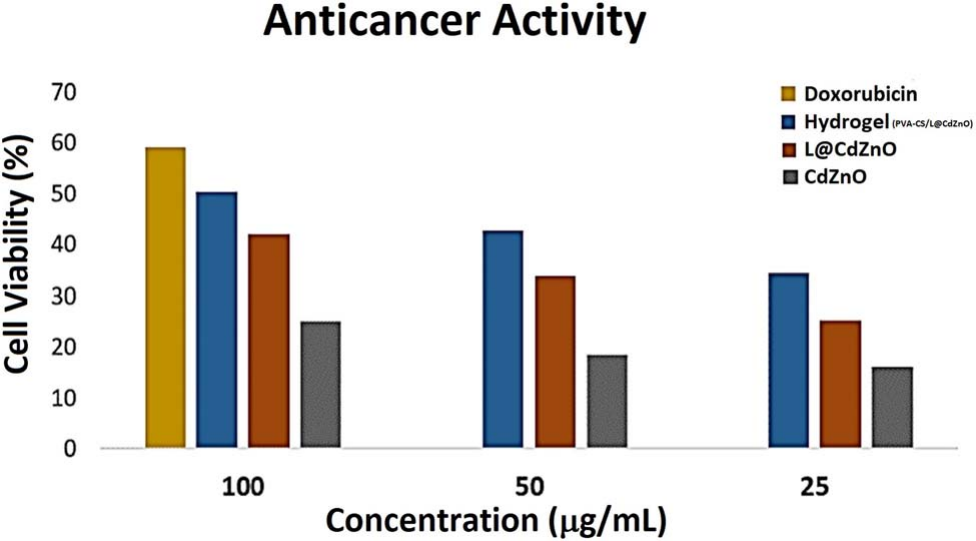
Anticancer activity of CdZnO NPs, L@CdZnO, and the PVA–CS/L@CdZnO hydrogel composite.

The anticancer activity of the PVA–CS/L@CdZnO hydrogel was assessed against MCF7 cell lines. Results revealed that the PVA–CS/L@CdZnO hydrogel, L@CdZnO, and CdZnO exhibited anticancer activity against the MCF7 cancerous cell line, where the IC50 values were 100.01, 100.34, and 99.78, respectively. Moreover, the anticancer activity of the PVA–CS/L@CdZnO hydrogel, L@CdZnO, and CdZnO towards MCF7 at a concentration of 100 μg mL^−1^ was 50.3%, 41.93%, and 25%, respectively, as shown in Fig. 9. CdZnO-, L@CdZnO-, and hydrogel-based systems generate free radicals that cause the formation of reactive oxygen species (ROS). By disrupting cellular metabolic processes and damaging DNA, proteins, and lipids, these ROS cause oxidative stress in cancer cells, which in turn causes apoptosis.^41,54^ On the basis of the reported evidences, Cd and Zn of PVA–CS/L@CdZnO hydrogel, specifically disrupt mitochondrial proteases, activating caspases to induce apoptosis and halting the cell cycle, thereby preventing further cancer cell proliferation.^55^ Recent studies more effectively present the smart hydrogel concept for effective cancer therapy.^56^

## 4 Conclusion

This study developed an eco-friendly and cost-effective approach for the synthesis of L@CdZnO and PVA–CS/L@CdZnO hydrogel using lignin, PVA and chitosan. The fabricated nanocomposites and hydrogel were characterized by UV-visible, FTIR, XRD, and TGA analyses, confirming their high purity. CdZnO and lignin@CdZnO particles exhibited hexagonal wurtzite shapes with sizes of 19.05 nm and 24.61 nm, respectively, while PVA–CS/L@CdZnO hydrogel displayed an amorphous structure. Antibacterial studies revealed that CdZnO, L@CdZnO, and the hydrogel had the potential for *S. aureus*, *E. coli*, and *B. subtilis* bacterial growth inhibition at 12 mm, 11 mm, and 18 mm, respectively. Regarding the antioxidant activities, the hydrogel showed the maximum of 89% free radical scavenging potential at the concentration of 1000 μg mL^−1^, while all other materials showed less free radical scavenging activity. In the *in vivo* study of wound healing, the hydrogel showed significant results, with 91% healing of the wound on day 10 compared with the control, which healed the wound by almost 61% on day 10. The biocompatibility and protective nature of PVA–CS/L@CdZnO hydrogel make it a suitable scaffold for wound healing, tissue engineering, and anticancer activities. The novelty and inexpensiveness of the method make it more attractive.

## Author contributions

H. A., S. A. R. N., and S. U. H. contributed equally in all fields of this research work such as conceptualization, methodology, supervision, writing original – draft, investigation, formal analysis, and supervision. X. F., Y. S., and M. S. N. contributed in conceptualization, investigation, resources, and writing – review and editing. A. R. and N. A. L. contributed in methodology, formal analysis, data interpretation, writing – review and editing. All authors have approved the manuscript for submission.

## Conflicts of interest

All the authors declare no conflict of interest.

## Data Availability

Data will be made available on request.
